# The assessment and predictive factors of posttraumatic stress and growth in mothers of young children with genetic conditions: A protocol for a longitudinal study

**DOI:** 10.1371/journal.pone.0323071

**Published:** 2025-11-12

**Authors:** Aleksandra Mariola Kołecka, Judyta Borchet, Łucja Bieleninik

**Affiliations:** Faculty of Social Sciences, Institute of Psychology, University of Gdansk, Gdansk, Poland; University of Botswana, BOTSWANA

## Abstract

**Introduction:**

Diagnosing a child’s disease is a traumatic event that impacts parents’ psychological well-being and mental health and is associated with burden. This protocol of a longitudinal study aims to observe posttraumatic stress levels in mothers of children with a genetic disease. It will also explore posttraumatic growth in these mothers and identify its key predictors.

**Materials and methods:**

The project is a sequential study that integrates both cross-sectional and longitudinal designs. The study will involve mothers of children aged 0–3 years who have been diagnosed with a genetic disease by a physician specialist before their first birthday. Two measurements will be taken with a 6-month interval between the first and second assessments. The study will use a questionnaire-based approach. The research assessment will be conducted using: the Impact of Event Scale-Revised (IES-R), the Posttraumatic Growth Inventory (PTGI), the Zimbardo Time Perspective Inventory (ZTPI), the Parental Burnout Assessment (PBA), the Kansas Inventory of Parental Perceptions (KIPP), and the Family Resilience Assessment Scale (FRAS).

**Discussion:**

This study may provide valuable insights into how mothers cope with a child’s genetic disease, both from a time perspective and a parental perspective. It could inform the development of targeted support strategies to help mothers manage the challenges of raising a child with a genetic disease, particularly in areas such as crisis intervention or therapy. We hypothesize that improving the mother’s time perspective could improve the mother’s well-being and thus enhance the overall functioning of the family system and support the child’s development.

## Introduction

### Background

Time perspective refers to how a person perceives and experiences time in their life. The perspective influences how individuals organize their experiences, make decisions, and interpret the world around them. Research by Zimbardo and Boyd (2008) suggests that time perspective is relatively stable, but stressful, uncontrollable, and unpredictable situations can alter a person’s time perspective, often narrowing their focus and making it difficult to maintain a broader view of time [1–4]. In some cases, it may even cause individuals to concentrate exclusively on one aspect of their time perspective (past, present, or future). Disease is one such event that can significantly change the way time is perceived [2,5–7].

Many studies have shown that patients with various somatic and mental diseases experience changes in their time perspective over time. The experience of disease significantly impacts the time perspective of patients with conditions such as cancer [3,8–12], multiple sclerosis [13], Alzheimer’s disease [14], renal failure [7], inflammatory bowel disease [15], depression [16], schizophrenia [17], and other mental health disorders [18]. Patients often become more focused on a negative past and fatalistic present compared to individuals without health conditions [3,4]. Additionally, they frequently experience a narrowing of their future time perspective [14].

Experiencing diseases can alter the time perspective not only of the patient but also of their caregivers and family members. Research has shown that caregivers and families of individuals with chronic diseases often notice significant changes in their perception of time [1–11]. Life becomes more unpredictable, and daily routines are disrupted. Caregivers frequently feel uncertain and anxious about the future, which can increase perceived stress [19]. Family members may experience time pressure and the constant need for support and care, leading to overload and fatigue [20]. The stress experienced by parents is especially heightened when a child is diagnosed with a chronic disease [21–29].

### Study rationale

The rationale for this study is to explore the experience of posttraumatic stress and posttraumatic growth in mothers of children with genetic diseases. Although there is a wealth of research on posttraumatic stress and posttraumatic growth in individuals with chronic disease [12–20], limited attention has been given to how these experiences manifest in parents, particularly mothers of children with genetic diseases. A child’s genetic disease constitutes an event characterized by high level of stress and unpredictability, which may influence the mother’s temporal perspective. Such conditions can hinder the ability to operate within the full temporal horizon, often resulting in a narrowed perception of time that emphasizes a singular temporal focus [21–24]. Additionally, the specific living circumstances engendered by genetic disease can induce alterations in individuals’ perception of time, reflecting adaptive or maladaptive changes in temporal cognition [22,25–27].

This research will fill a critical gap in understanding how a child’s genetic disease impacts family life and provide evidence-based recommendations for supporting mothers and families coping with this challenging situation. The impact of mothers’ adopted temporal perspectives on their coping strategies may represent a valuable therapeutic resource in clinical settings.

### Objectives and hypotheses

The study seeks to provide valuable insights into how mothers navigate the experience of having a child with a genetic disease, both from a time perspective and a parenting perspective. We hope that this will lead to a deeper understanding of the mothers’ situation, their behaviors, and their attitudes toward time. The variables analyzed will be: the intensity of posttraumatic stress, the level of posttraumatic growth, the mother’s time perspective, parental burnout, the perception of the mother’s experiences related to parenthood, and family resilience.

The main objective of this study is to verify the time perspective adopted by mothers experiencing a child’s genetic disease. It will answer the research question: Does the mother’s time perspective change significantly during the experience of a child’s genetic disease?

Specific objectives are as follows:

1. to explore the phenomenon of posttraumatic stress in the mother of a child with a genetic disease in the context of her time perspective
2. to explore the phenomenon of posttraumatic stress in the mother of a child with a genetic disease from a parental perspective
3. to explore the phenomenon of posttraumatic growth in the mother of a child with a genetic disease in the context of her time perspective
4. to explore the phenomenon of posttraumatic growth in the mother of a child with a genetic disease from a parental perspective
5. to indicate predictors of posttraumatic stress in the mother of a child with a genetic disease
6. to indicate predictors of posttraumatic growth in the mother of a child with a genetic disease

We hypothesize in this study that:

1. The mother’s time perspective changes during the experience of the child’s genetic disease [22,23,25–28].
2. Time perspective is associated with the intensity of posttraumatic stress in the mother of a child with a genetic disease: a negative past time perspective increases the intensity of posttraumatic stress in the mother of a child with a genetic disease; a positive past time perspective decreases the intensity of posttraumatic stress in the mother of a child with a genetic disease; a present hedonistic time perspective decreases the intensity of posttraumatic stress in the mother of a child with a genetic disease; a present fatalistic time perspective increases the intensity of posttraumatic stress in the mother of a child with a genetic disease; a positive future time perspective decreases the intensity of posttraumatic stress in the mother of a child with a genetic disease [21–24,29,30].

Traumatic events might affect one’s time perspective, make it difficult to act in the full time horizon, and often limit the perception of time to an increased concentration on one of the time perspectives [21–24]. The disease shapes specific living conditions that can generate changes in the perspective of time perception [22,25–27]. So far, a significant impact of the experience of illness on the time perspective has been demonstrated in patients with: cancer [23,31–35], multiple sclerosis [36], Alzheimer’s disease [29], renal failure [27], inflammatory bowel disease [30], depression [37], schizophrenia [38], as well as other mental illnesses [39]. Typically, people experiencing illnesses were more focused on the negative past and fatalistic present than control participants [23,24]. Also, narrowing of the future time perspective was often observed [29].

1. Parental burnout is positively associated with the intensity of posttraumatic stress in the mother of a child with a genetic disease [6,40–44].

There is no evidence for a direct relationship between parental burnout and PTSD in parents of children with chronic illness. However, it is known that parents of children with chronic diseases are at increased risk of parental burnout [40–44]. Parental burnout is a response to long-term parenting stress [45], is associated with physical and emotional exhaustion, and may pose a potential risk for the development of posttraumatic stress disorder. The intensity of parental burnout has been observed, among others, in parents of children [45–47] and adults with autism spectrum disorder [47], children with special needs [48], neurodevelopmental disorders [49], type I diabetes [42], cancer [50], as well as in mothers of children with ADHD [51], rare genetic disease [6] and in mothers of children with complex medical problems [52].

1. Family resilience is negatively associated with the intensity of posttraumatic stress in the mother of a child with a genetic disease [53–59].

Research emphasize the importance of family resilience in the process of effectively coping with stress experienced by parents, for example in parents of children with mental, emotional and behavioral disorders [54], asthma [53], type 1 diabetes [57], cancer [59], and coronary artery disease [55,56]. Family resilience may be a protective factor against the dynamics of posttraumatic stress [58]. At the same time, experiencing a difficult situation, such as a child’s illness, poses a threat to family resources and may, in turn, cause a decrease in family resilience. Studies show that families with children affected by chronic diseases may exhibit low levels of family resilience [60,61].

1. Time perspective is associated with the level of posttraumatic growth in the mother of a child with a genetic disease: a negative past time perspective decreases the level of posttraumatic growth in the mother of a child with a genetic disease; a positive past time perspective increases the level of posttraumatic growth in the mother of a child with a genetic disease; a present hedonistic time perspective increases the level of posttraumatic growth in the mother of a child with a genetic disease; a present fatalistic time perspective decreases the level of posttraumatic growth in the mother of a child with a genetic disease; a positive future time perspective increases the level of posttraumatic growth in the mother of a child with a genetic disease [30,62–67].

There is no evidence for a direct relationship between the parental time perspective and the level of posttraumatic growth in the parent of a child with a chronic disease. However, studies have shown that the future time perspective is among the predictors of posttraumatic growth in young adults [62]. A genetic disease in a child requires the reorganization of the everyday life of the entire family system, directing the parent’s activities to provide optimal support for the development of the child with a genetic disease, which is associated with effort spread over a long time perspective. Based on the above, it is assumed that a positively valued future, concentration on long-term goals, and current support activities may turn out to be a factor contributing to the occurrence of posttraumatic growth in the mother of a child with a genetic disease.

1. Family resilience is positively associated with the level of posttraumatic growth in the mother of a child with a genetic disease [53–59,68–70].

Resilience allows the family to adapt to various difficulties or traumatic experiences [70]. Many studies emphasize the importance of family resilience in the process of adaptation to a child’s illness, including in the case of families with children with mental, emotional and behavioral disorders [54], asthma [53], type 1 diabetes [57], cancer [59], and coronary artery disease [55,56]. Family resilience has been shown to support positive ways of coping with a child’s chronic illness and to develop stronger relationships, better family cohesion, and a positive family belief system [68]. High family resilience is an important factor for posttraumatic growth [71,72]. Family-related resources increase the chances for an upward trajectory of posttraumatic growth [58,69].

## Materials and methods

A study protocol has been developed in accordance with the SPIRIT guidelines (see annex). The project received ethics approval from The Research Ethics Board at the University of Gdansk (approval number: 50/2024/WNS, approval date: 23 June 2024).

### Study design

**Fig 1:**
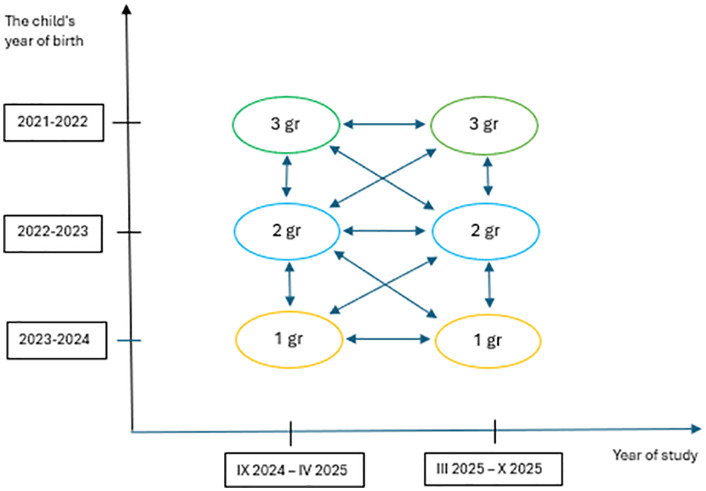
Research design diagram.

The project is a sequential study that integrates both cross-sectional and longitudinal designs. Two measurements are planned, with a 6-month interval between the first and second assessment. The first participant is expected to be enrolled in the study in September 2024, and the last participant in April 2025. Each undergoes a follow-up assessment 6 months after the initial measurement. The second measurement is scheduled to take place between March 2025 and October 2025 (Fig 1).

### Study procedure

Referrals will be made by a principal investigator, who will provide both oral and written explanations and descriptions of the project. Participants will be informed that enrollment is voluntary and that they may withdraw from the study at any time. Recruitment will be based on the eligibility criteria established for the study group. Each participant will receive detailed information about the study’s objectives and procedures. After obtaining informed consent, an interview will be conducted to collect socio-demographic data. Following this, the mother will complete the initial set of questionnaires (baseline assessment).

**Fig 2:**
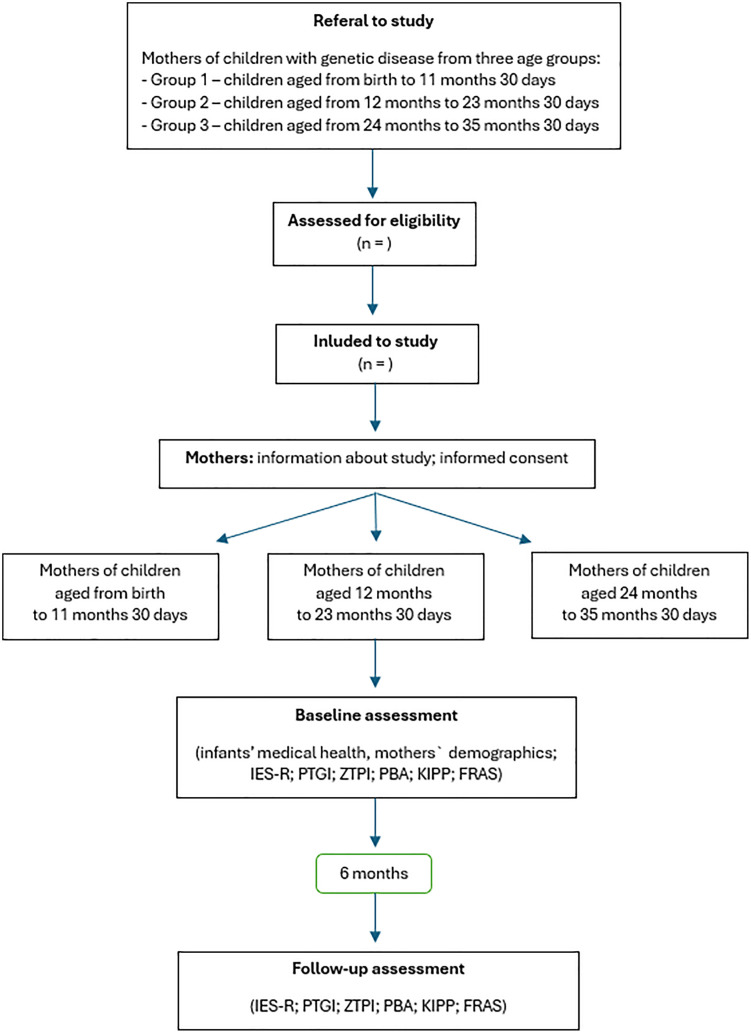
Stages of the research. IES-R: Impact of Event Scale Revised (Juczyński & Ogińska-Bulik, 2009), PTGI: Posttraumatic Development Inventory (Ogińska-Bulik & Juczyński, 2010), ZTPI: Zimbardo Time Perspective Questionnaire (Przepiórka, 2011), PBA: Parental Burnout Questionnaire (Szczygieł et al., 2020), KIPP: Kansas Inventory of Parental Perceptions (Pisula & Noińska, 2020), FRAS: Family Resilience Assessment Scale (Nadrowska et al., 2021).

After the questionnaires are completed, a follow-up meeting will be scheduled approximately six months later. During the second meeting, the mother will complete the same set of questionnaires (2nd assessment) (Fig 2). Each study participant will receive feedback on the study results. Furthermore, each mother will be presented with an opportunity for a psychological consultation with a psychologist experienced in working with people with disabilities and their families.

Each participant may withdraw from the study at any time without providing a reason. Withdrawal can be communicated directly to the principal investigator via in-person, telephone, or email contact. If the mother is unable to participate in the second measurement, she will be excluded from the study.

### Study participants and study settings

The study will involve mothers of children aged from birth to 3 years diagnosed with a genetic disease: cystic fibrosis (CF), spinal muscular atrophy (SMA), or another genetic disease diagnosed in the child before the age of 1. The project will have a multicenter design. Through the study, we aim to include mothers from various voivodeships across Poland in order to enhance the generalizability of the findings. Eligibility for the study will be based on the inclusion criteria presented below. The child’s diagnosis was confirmed through genetic testing. Information regarding the diagnosis was provided to the researcher by the attending physician, including specialists such as a pediatrician, geneticist, neonatologist, or cardiologist.

The participants will be grouped according to the age of their children into the following categories:

- Group 1 – children aged from birth to 11 months 30 days,
- Group 2 – children aged from 12 months to 23 months 30 days,
- Group 3 – children aged from 24 months to 35 months 30 days.

### Eligibility criteria

#### Inclusion criteria.

The study will include **mothers.** The rationale for targeting only mothers, rather than both parents, is based on the significant role mothers often play in early child caregiving, especially in supporting children diagnosed with genetic diseases, as mothers usually are their primary caregivers [73–77]. **The study will include mothers of children diagnosed with a genetic disease before the age of 1**. The rationale for including only mothers of children diagnosed before the age of 1 year is to allow for meaningful comparisons between the study groups. The criterion of early diagnosis of the child will ensure that each mother will experience the diagnosis of the child’s genetic disease at a similar time in the child’s life. This will make the groups more homogeneous in terms of the timing of the critical event. This limitation will take into account the initial stages of adaptation to the genetic diagnosis, ensuring that the results reflect the unique challenges and stressors experienced by mothers in the time since their child’s diagnosis.

The child should be diagnosed by a specialist physician with one of the following genetic diseases: cystic fibrosis (CF), spinal muscular atrophy (SMA), or other genetic disease. The motivation for focusing on these diagnoses stems from consultations with medical personnel. Among all congenital diseases detected in Poland through prenatal testing of newborns, only CF and SMA are genetically determined. In contrast, other conditions may be temporary diagnoses (such as congenital hypothyroidism, hearing defects). Additionally, some congenital metabolic disorders, like phenylketonuria and biotinidase deficiency, can often be managed with appropriate treatment strategies and elimination diets, which may mitigate or eliminate symptoms of the disease.

The child must be **under the age of 3.** The rationale for including children up to 3 years of age is based on the critical developmental period that occurs during the first three years of life. This stage is characterized by rapid physical, cognitive, and emotional growth, during which children often experience the initial impact of a genetic disease. The early years are also a time when mothers experience the novelty of this challenging situation, are highly involved in the care of a child with a genetic disorder, and are coping with their health-related challenges. Furthermore, taking into account the periodization of child development and limiting the age of the child to early childhood will ensure better generalization of the results [78,79].

#### Exclusion criteria.

Having another child with a genetic or chronic disease born earlier. We have decided to exclude mothers who have more than one child with a genetic disease or a chronic disease due to the possible confound related to their previous experiences. These mothers may have already developed their coping strategies for dealing with child diagnosis. This could result in different adaptive patterns, which may not be representative of the initial response to a child’s first genetic diagnosis. As a result, these mothers might exhibit a different intensity of posttraumatic stress or a changed level of posttraumatic growth. Research suggests that repeated exposure to the same type of traumatic event can lead to increased psychological stability and changes in mental resilience, as well as variations in the intensity of post-traumatic stress [30]. Additionally, a mother’s perception of time may shift based on the experience gained from previous crisis situations [6].

Diagnosis of lethal developmental defects in a child. Mothers of children diagnosed with lethal defects were excluded from the study due to the unique nature of the situation, where the child’s disease ultimately leads to death. The death of a child poses a significant threat to the parenting role and requires specialized support [80]. Parenting during a child’s terminal disease involves interventions tailored to this specific experience, which focus on helping parents cope with the impending loss. These interventions include psychological support aimed at acknowledging the parenthood experience, creating positive memories with the child, preparing for the child’s death, and helping parents adjust to a new family dynamic after the loss [81]. The diagnosis of lethal defects often requires palliative care interventions [33].

If it is impossible to include the mother in measurement II, despite applying the intention to treat approach, the mother will be excluded from the study.

### Outcomes

**The Intensity of the Posttraumatic Stress Symptoms** in mothers will be measured using the Revised Impact of Events Scale (IES-R, Weiss & Marmar, 1997; the Polish adaptation: Juczyński & Ogińska-Bulik, 2009). The scale consists of 22 statements that assess stress symptoms over the past seven days in relation to the experienced traumatic event. It evaluates three dimensions of posttraumatic stress: Intrusion, Arousal, and Avoidance. Responses are rated on a five-point Likert scale (from “0” - “not at all” to “4” - “definitely yes”). Scores are calculated for the overall scale and for individual dimensions. The results reflect the intensity, rather than the frequency of symptoms experienced by the participant. The cut-off value is set at 1.5 points, which applies to both the general PTSD score and its individual dimensions. Scores exceeding this cut-off value indicate at least moderate posttraumatic stress. A higher score corresponds to greater severity of PTSD symptoms. The tool has achieved satisfactory psychometric properties with internal consistency (Cronbach’s alpha) of.92 for the overall scale, and.89,.85, and.78 for Intrusion, Arousal, and Avoidance, respectively [34].

**The level of the post-traumatic growth** will be measured using the Posttraumatic Growth Inventory (PTGI, Tedeschi & Calhoun, 1996; the Polish adaptation: Ogińska-Bulik & Juczyński, 2010). The inventory consists of 21 statements that describe positive changes resulting from a negative and traumatic life event. Participants rate each statement on a six-point scale, from “0” (indicating no change as a result of the crisis) to “5” (indicating a very large extent of change). The inventory assesses four factors of posttraumatic growth: Changes in self-perception, Changes in relationships with others, Greater appreciation of life, and Spiritual changes. An overall score and individual factor scores are calculated. The overall score is the sum of all the above-mentioned factors, with higher scores indicating greater positive change and, therefore, a higher level of posttraumatic growth. The tool demonstrates satisfactory psychometric properties with a Cronbach’s alpha coefficient of.93 for the overall scale and individual factors ranging from.63 to.87) [35].

### Predictors

**The time perspective** will be measured using the Zimbardo Time Perspective Inventory (ZTPI, Zimbardo & Boyd, 1999; the Polish adaptation: Przepiórka, 2011). The inventory consists of 56 statements and assesses the attitude towards time across five dimensions: Present hedonism, Present fatalism, Positive past, Negative past, and Future. Participants rate their agreement with each statement on a five-point scale, where “1” means “I completely disagree” and “5” means “I completely agree”. Scores are calculated for each dimension, reflecting the individual’s subjective attitude toward each time perspective. All scales demonstrate satisfactory internal reliability with Cronbach’s alpha coefficient for individual dimensions as follows: Negative past (α = .84); Positive past (α = .80); Present hedonism (α = .82); Present fatalism (α = .70); Future (α = .82) [36–37].

**The parental burnout** will be measured using the Parental Burnout Questionnaire (PBA, Roskam et al., 2017; the Polish adaptation: Szczygieł et al., 2020), which consists of 23 questions assessing four dimensions of parental burnout: Exhaustion with the parental role, Contrast with the previous image of oneself as a parent, Loss of pleasure in being a parent/Saturation with the parental role, and Emotional distancing from the child. The frequency of burnout-related feelings is rated on a seven-point Likert scale ranging from “0” (never) to “6” (every day). Scores are calculated for the overall scale and for each individual dimension. The tool demonstrates excellent internal consistency, with a Cronbach’s alpha of.96 [38]. **The perception of parenting experiences** will be measured using the Kansas Inventory of Parental Perceptions (KIPP, Behr et al., 1992; the Polish adaptation: Pisula & Noińska, 2020). This tool consists of four scales: Positive child influence, Social comparisons, Search for cause, and Sense of control. Participants respond to individual items within each scale by selecting one of four response options: “definitely not”, “no”, “definitely yes” and “yes”. Scores are calculated for each of the four scales. To date, no Polish studies have examined the factor structure, validity and reliability of the KIPP scales. In the original version, Cronbach’s alpha coefficients are.77 for the “Positive child contribution” scale, 0.66 for the “Social comparisons” scale,.87 for the “Search for cause” scale and.79 for the “Sense of control” scale [39]. **The family resilience** will be examined by the Family Resilience Assessment Scale (FRAS, Walsh, 1996; the Polish adaptation: Nadrowska et al., 2021). This tool consists of 54 items across six subscales, which evaluate various aspects of family resilience. The subscales assess the following areas: Family communication and problem-solving, Use of social and economic resources, Maintaining a positive attitude, Family ties, Family spirituality, and Ability to give meaning to adversity. Each item is rated on a four-point scale, with the following response options: “strongly agree”, “agree”, “disagree”, “strongly disagree”. Higher scores indicate a higher level of family resilience. The internal reliability of the subscales is satisfactory, with a Cronbach’s alpha coefficient of.96 for the entire scale [40].

Furthermore, the following sociodemographic data will be collected:

1. mother’s age [in years],
2. education level [primary, secondary vocational, secondary technical, secondary general, higher bachelor’s degree, higher master’s degree],
3. place of living [village, town with a specified number of inhabitants],
4. marital status [single, married, informal union, divorced, separated, widow],
5. employment status [employed under an employment contract, employed under a civil law contract, own business, student, housewife, unemployed and looking for work, unemployed due to health condition, other],
6. economic situation [very good, good, average, poor, bad],
7. information on additional sources of income, benefits received [care benefits, emergency allowances, financial assistance from the family, foundation assistance, alimony, other],
8. estimated average monthly costs related to the child’s treatment and rehabilitation [in PLN],
9. number of children,
10. as well as the information about the child with a genetic disease [age, gender, birth order, and specific genetic diagnosis].

### Power calculation and sample size

Based on data provided by the European Platform on Rare Disease Registration (2025) [82], between 2013 and 2023, the average prevalence of births of children with anomalies, including genetic anomalies, was 2.7%. As the literature on the prevalence of genetic diseases diagnosed in children before the age of 1 in Poland is limited and the Polish healthcare system faces diagnostic challenges [83], we used the European indicator.

To compute the necessary sample size, the G*Power 3.1.9.7 program was used. The assumptions used for the calculation were: two tails, effect size g = 0.3, an alpha error of.05, power of 85%, and a constant proportion of.27. The estimated sample size for each birth year was 34. In the current study, mothers who gave birth across three different calendar years will be included; thus, the number of study participants is estimated as 102.

To achieve the target sample size (N = 102), participants will be recruited from various early intervention centers, hospitals, associations, and foundations supporting parents and their children with genetic diseases. Recruitment will focus on centers providing treatment and rehabilitation for children in Poland. Considering a 15% loss to follow-up, the final sample will be 120 mothers.

Therefore, 40 participants will be recruited for each of the study groups:

- Group 1 – children aged from birth to 11 months 30 days,
- Group 2 – children aged from 12 months to 23 months 30 days,
- Group 3 – children aged from 24 months to 35 months 30 days.

### Statistical methods

Descriptive analysis will be applied to qualitative variables, presenting both absolute and relative frequencies. For quantitative data, central tendency and dispersion measures will be calculated, depending on the distribution assessed using the Kolmogorov-Smirnov test. Relationships between variables will be examined through Pearson’s correlation coefficient and regression analysis. To compare results between measurement points I and II, either the paired Student’s t-test or its non-parametric alternative will be used. Dependent variables Results will be considered statistically significant when a *p*-value of less than .05 is observed. Statistical analysis will be performed using SPSS STATISTICS 29.

If missing data (e.g., due to attrition) occurs, given the sample size, for cross-sectional comparisons, the multiple imputation technique will be employed. For the longitudinal (cohort) component, analyses will follow a modified intention-to-treat principle, where participants with at least baseline data are retained in the analytic sample [84,85].

### Data management

The data collected in the study, including information about both the mother and the child, will remain confidential. No personal data will be disclosed to the public. Only the researchers conducting the study will have access to the data, and the results will be presented in aggregate form. Participant data will be anonymized to ensure that it cannot be linked to identifiable personal information. Personal data will not be published and will be securely stored in a locked cabinet within the premises of the University of Gdansk. Each participant will be assigned a unique identifier number to enable accurate linkage of data collected at baseline and follow-up. The anonymized datasets will be electronically archived for potential use in future research. Paper records will be securely destroyed upon completion of the project.

## Discussion

We intend to explore whether the mother’s time perspective changes significantly during the experience of a child’s genetic disease. In this study, we take into account the periodization of child development. It included mothers of children with a genetic disease in early childhood, i.e., from birth to 3 years of age [41,42], as the sample homogeneity allows for better generalization of the results. The sequential nature of the study allows for the observation of the dynamics of changes in posttraumatic stress and posttraumatic growth in mothers of children with genetic disease in a broader timeframe, while measurements from several time points are obtained at the same time.

The study will significantly contribute to the development of the discipline by exploring the importance of time perception in the process of mothers’ coping with the situation of the child’s genetic disease. Although there are scientific reports on the role of time perspective on the intensity of posttraumatic stress and the level of posttraumatic growth, there is a gap in the scope of research on the relationship between the parent’s time perspective and the intensity of posttraumatic stress and the level of posttraumatic growth in the parent in the situation of the child’s genetic disease, while taking into account parental factors and the dynamics of changes occurring during the child’s disease. The posttraumatic stress rates studied in the group of mothers of children with genetic diseases will allow for quick intervention and providing the necessary psychological support to mothers by providing them with consultations and referring them to institutions providing specialist help.

The results of the study may constitute a solid theoretical basis for further experimental studies towards the use of elements of time perspective balancing therapy as a method of short-term therapeutic intervention for mothers in the first moments after receiving a diagnosis of a child’s genetic disease. Using mothers’ awareness of the impact of their time perspective on the ways of coping with a crisis situation may prove to be a potential therapeutic tool in clinical work.

However, the study has its limitations. Possible limitations of the study may include: a low retention rate and limited access to the study group. Analysis of the results will be possible if the mother participates in both study measurements. If it is not possible to include the mother in the second measurement, the person will be excluded from the study. Due to the specificity of genetic diseases, which belong to the group of diseases with limited incidence, reaching the desired sample size may be difficult.

Another important limitation of this study is the exclusive focus on mothers of children with genetic diseases. This decision was primarily due to the insufficient number of fathers being present at the recruitment settings, which would not allow for enrollment of a sample large enough for reliable statistical analysis. Consequently, this limits the generalizability of the study findings to mothers only. Fathers, who often play both primary and secondary caregiving roles, may experience different patterns of PTSD symptoms, parental burnout, and posttraumatic growth. Therefore, future research should aim to include a more gender-diverse sample of parents to provide a more comprehensive understanding of the psychological processes within families affected by genetic diseases.

Another limitation of the proposed protocol might be the choice of a 6-month follow-up period. Although this timeframe allows for the observation of many acute posttraumatic stress responses and early indicators of posttraumatic growth [86,87] it may not be sufficient to capture the full development and long-term trajectory of posttraumatic features. The length of posttraumatic processes can vary significantly between individuals, with some symptoms and growth experiences emerging or evolving well beyond six months. The 6-month period was selected to balance capturing meaningful psychological changes with practical considerations such as participant retention and study feasibility. Nonetheless, future research should consider longer follow-up durations to gain a more comprehensive understanding of the evolution of posttraumatic symptoms and growth over time.
